# Automatically Characterizing Sensory-Motor Patterns Underlying Reach-to-Grasp Movements on a Physical Depth Inversion Illusion

**DOI:** 10.3389/fnhum.2015.00694

**Published:** 2016-01-05

**Authors:** Jillian Nguyen, Ushma V. Majmudar, Jay H. Ravaliya, Thomas V. Papathomas, Elizabeth B. Torres

**Affiliations:** ^1^Graduate Program in Neuroscience, Rutgers UniversityPiscataway, NJ, USA; ^2^Department of Biomedical Engineering, Rutgers UniversityPiscataway, NJ, USA; ^3^Center for Cognitive Science, Rutgers UniversityPiscataway, NJ, USA; ^4^Department of Psychology, Rutgers UniversityPiscataway, NJ, USA; ^5^Department of Computer Science, Rutgers UniversityPiscataway, NJ, USA

**Keywords:** statistical platform, sensory-motor integration, visuomotor behavior, action and perception, visual illusions, visuomotor integration

## Abstract

Recently, movement variability has been of great interest to motor control physiologists as it constitutes a physical, quantifiable form of sensory feedback to aid in planning, updating, and executing complex actions. In marked contrast, the psychological and psychiatric arenas mainly rely on verbal descriptions and interpretations of behavior via observation. Consequently, a large gap exists between the body's manifestations of mental states and their descriptions, creating a disembodied approach in the psychological and neural sciences: contributions of the peripheral nervous system to central control, executive functions, and decision-making processes are poorly understood. How do we shift from a psychological, theorizing approach to characterize complex behaviors more objectively? We introduce a novel, objective, statistical framework, and visuomotor control paradigm to help characterize the stochastic signatures of minute fluctuations in overt movements during a visuomotor task. We also quantify a new class of covert movements that spontaneously occur without instruction. These are largely beneath awareness, but inevitably present in all behaviors. The inclusion of these motions in our analyses introduces a new paradigm in sensory-motor integration. As it turns out, these movements, often overlooked as motor noise, contain valuable information that contributes to the emergence of different kinesthetic percepts. We apply these new methods to help better understand perception-action loops. To investigate how perceptual inputs affect reach behavior, we use a depth inversion illusion (DII): the same physical stimulus produces two distinct depth percepts that are nearly orthogonal, enabling a robust comparison of competing percepts. We find that the moment-by-moment empirically estimated motor output variability can inform us of the participants' perceptual states, detecting physiologically relevant signals from the peripheral nervous system that reveal internal mental states evoked by the bi-stable illusion. Our work proposes a new statistical platform to objectively separate changes in visual perception by quantifying the unfolding of movement, emphasizing the importance of including in the motion analyses all overt and covert aspects of motor behavior.

## Introduction

Our bodies are in constant motion. Whether motor acts are under voluntary control, or occur largely beneath awareness, movement variability is inherently present within natural behaviors (Bernstein, 1967). The naked eye is incapable of detecting all aspects of movements. In the words of Nikolai Bernstein “The movements of the body are too fast and fleeting to be captured by the ordinary eye.” The human brain must select, prioritize, and integrate a vast amount of information from multiple sensory modalities to interact and rapidly adapt to our environments (Mesulam, 1998). We as observers simply cannot analyze the continuous flow of movement in detail. We are only able to subjectively detect and interpret unambiguous features of our motor actions, while other motions supplementing goal directed behavior go largely unnoticed (Torres, 2011). Often studies of movement focus exclusively on discrete segments of goal-directed behavior and leave out other ambiguous segments, possibly obscuring significant contributions of the sensory-motor system to our understanding of intended behavior. Such ambiguous segments that coexist with goal-directed ones are physically quantifiable. Indeed, what we may treat as a nuisance in our data often contains a wealth of information about decisions and actions (Torres et al., 2013). Furthermore, what we may call noise may contain valuable signal.

Objective assessments of natural behaviors and inference about our mental states are made possible through the measurement of movement kinematics. For example, contemporary approaches to embodied cognition aiming at inferring intention from action (Torres, 2010; Becchio et al., 2014) have built a body of evidence on this topic (Gilden and Proffitt, 1989; Gilden, 1991). Other areas of movement research highlight the importance of studying movement variability as a physical, quantifiable form of sensory feedback that is imperative to the planning, on-line control, and execution of complex actions (Bernstein, 1967; van Beers et al., 2002, 2004; Torres et al., 2014). Along these lines, numerous studies of motor control quantify and model the kinematics of movement trajectories during goal-directed behaviors. Some focus on statistical features of endpoint errors around targets during pointing tasks (Harris and Wolpert, 1998). Others focus on joint angles variability (Latash et al., 2001; Torres et al., 2011; Dutta et al., 2013) or speed variability (Churchland et al., 2006; Torres and Andersen, 2006), or the variability of geometric quantities tied to muscle synergies (d'Avella et al., 2003; Todorov, 2004, 2005, 2009), among others.

**Figure 1:**
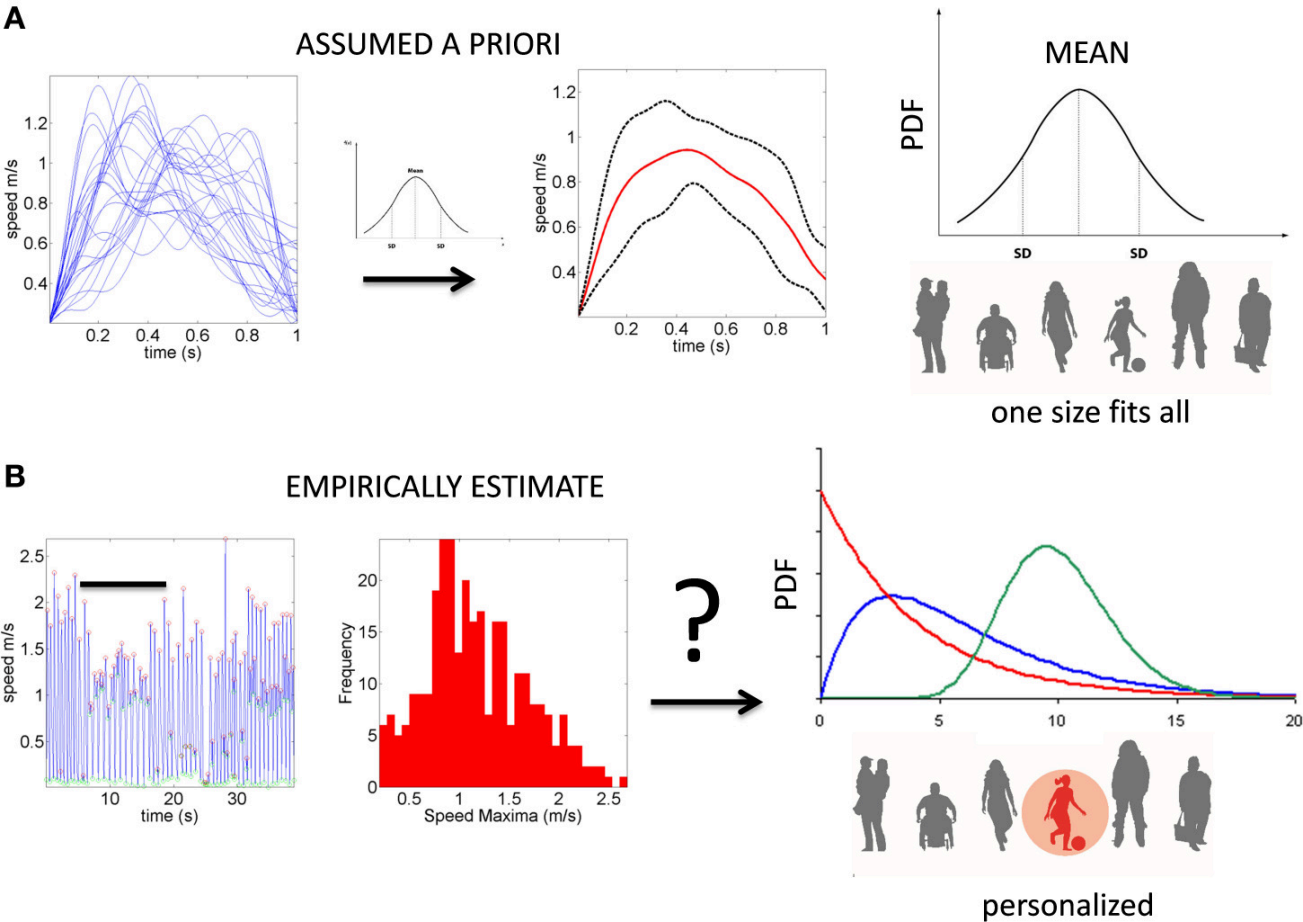
**Two different statistical approaches to the same set of kinematic parameters**. **(A)** Traditional approaches to assess motor variability follow a “one-size-fits-all” method. Data from movements (e.g., a kinematic, speed-dependent variable) is averaged across epochs of experimental trials under an assumed theoretical symmetric distribution such as the Gaussian distribution and examined under parametric models that assume additive statistics. **(B)** New proposed statistical platform for the personalized stochastic profiling of the participant. In this approach the continuous data is examined and the motor fluctuations in performance assessed to determine their rate of accumulation along with the shifts in the empirically estimated parameters spanning a family of probability distributions for each person as a function of context, percepts, etc. The noise-to-signal ratios in this case are not assumed from theoretical parameters but rather estimated from physical data. Bar indicates the trials in **(A)** as embedded in the continuous flow of the behavior.

Despite the interest in movement variability and the body of knowledge that this interest has generated, the general statistical approach involving analyses of experimental movement data and/or movement models use a “one-size-fits-all” paradigm (Figure 1). Specifically, it is often assumed that the Gaussian probability distribution function (PDF) is appropriate to assess the motion data. This has been done without establishing how or if the PDF changes with perceptual processing, context or simply as people typically age. At the experimental level, such analyses often rely on epochs of kinematics data gathered and averaged across a handful of trials once the participant is over practiced (Figure 1A). In this sense current accounts of motor variability speak of fluctuations around an *assumed* theoretical mean (Harris and Wolpert, 1998; Hartung et al., 2005; Króliczak et al., 2006). Consequently, we know very little about empirically estimated PDF's from the actual data in the context of perceptual tasks.

**Figure 2:**
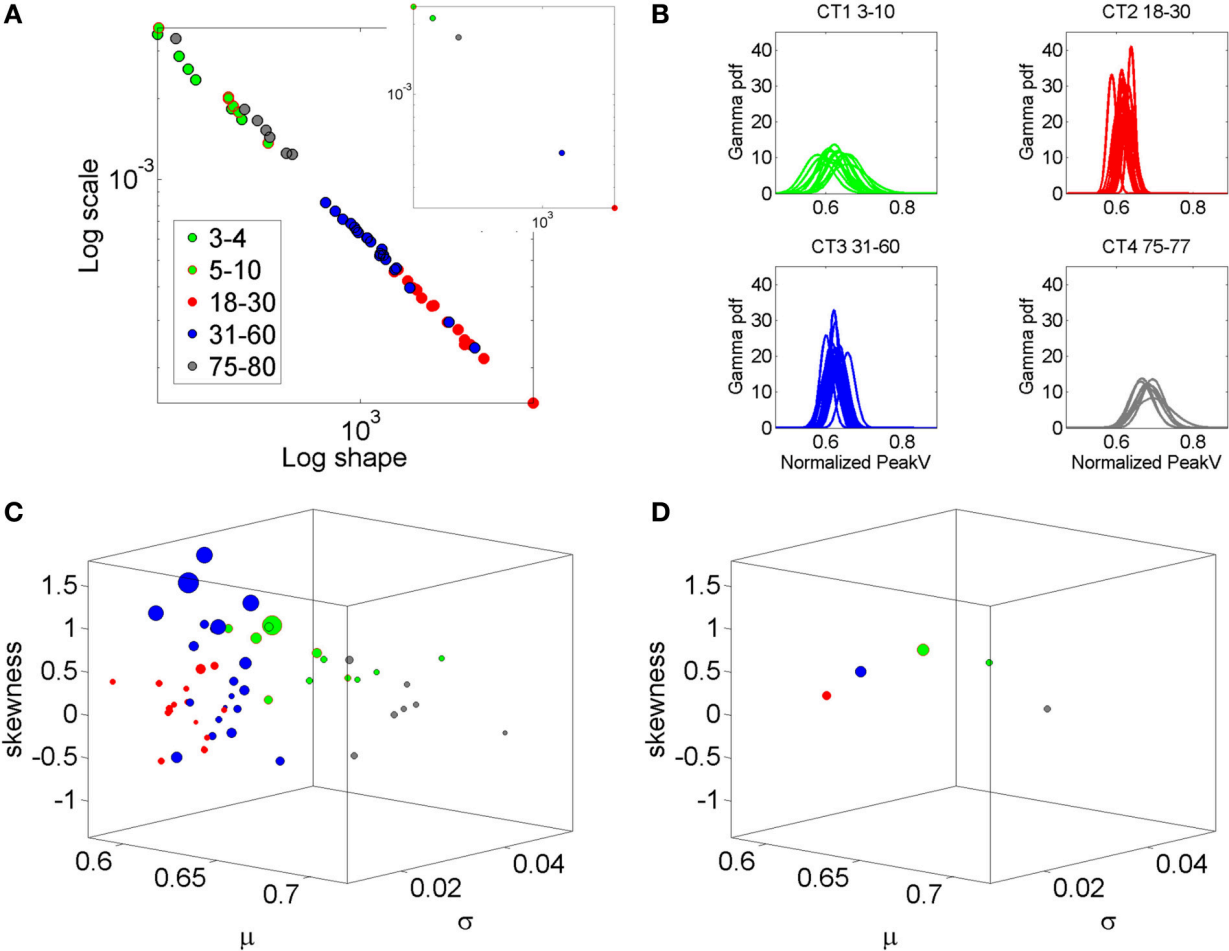
**Why a personalized approach is needed: Shift in stochastic signatures of velocity dependent parameters in a cross section of the human population across typical development and aging**. The parameter of interest is the normalized peak linear velocity index (see text for explanation). **(A)** The empirically estimated Gamma parameters of 76 participants color coded by age (see legend) whereby the young developing children have the highest noise to signal ratio and the most skewed distributions. As the age increases the young adolescents and young adults shift values toward Gaussian shapes and lower dispersion, but the noise increases in the elderly and the speed decreases. Inset shows the averaged values across each age group arbitrarily selected to show the relevance of estimating rather than assuming homogeneous PDF and mixing across ages (as it is often done). **(B)** Empirically estimated PDFs. **(C)** The individualized summary statistics estimated across the four moments, mean (X-axis), variance (Y-axis), skewness (Z-axis), and kurtosis (size of the marker). Notice that the young adolescents and young adults (red) are close to the zero-skewness level and have kurtosis 3 denoting Gaussian distribution but these values change for older adults and for the elderly. Young children have the highest variability as their system transitions into mature adulthood states. **(D)** Mean values to show the shifts across the groups.

In recent years we have deployed a research program aimed at personalized statistical analyses for Precision Medicine (Torres et al., under revision). The findings from this new platform for clinical research can be translated to basic perceptual science. In this paper we apply the new analytics to data from visual illusion experiments performed by typical controls. First we show the family of PDF's illustrating the summary statistics of the typical population at large alerting us that the Gaussian assumption is not appropriate in general (Figure 2). More precisely, by examining the stochastic motor signatures of a cross section of different developmental stages from 3–80 years of age this motivational graph demonstrates the evolution of the PDF's across the human lifespan. We use the present data from a complex illusion-driven task to illustrate the potential application of the new statistical platform to perceptual tasks.

We posit that self-produced movements, whether goal-directed or not, contribute to the continuous re-afferent stream of kinesthetic sensory feedback (Von Holst and Mittelstaedt, 1950; Von Holst, 1954; Reichenbach and Diedrichsen, 2015). In this sense, some segments in the motions are deliberate and occur with a clear purpose in mind while others are rather automatic or spontaneous in nature (Torres, 2011). They occur largely beneath the person's awareness but can provide a window into the person's levels of intent. In the recent past we have characterized the stochastic signatures of these supplementary motions in complex sports routines and simpler biomechanical pointing movements. Here we aim at better understanding the shift in signatures of such spontaneous segments in the context of deliberate reach-to-grasp movements as the hand shapes and orients to match the orientation of an illusory percept, or to do so when the percept is veridical. In the context of this illusion experiment, we provide a new statistical characterization of the fluctuations in performance and their statistical accumulation across movements with different levels of intent. We discover that those retraction movements that the participants were reportedly least aware of unambiguously revealed the illusory or veridical mental states of the participants.

## Materials and methods

### Rationale of experimental paradigm

**Figure 3:**
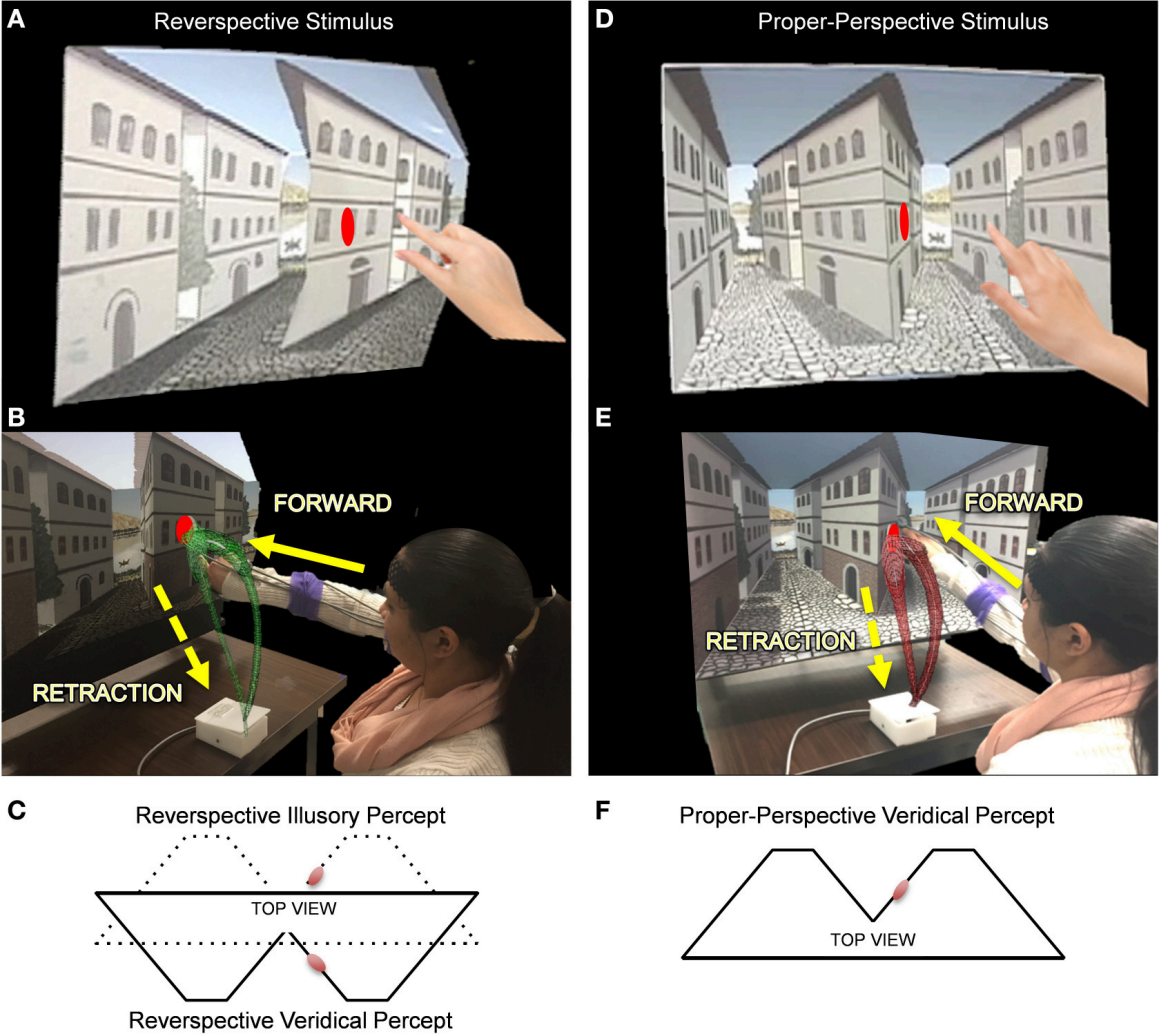
**The Reverspective Depth Inversion Illusion (DII) and the proper-perspective stimulus**. **(A,D)** Physical reverspective stimulus and the proper-perspective stimulus, respectively shown with their target locations. **(B,E)** Participant reaches forward toward the stimulus (in green and red, respectively) and then spontaneously (without instructions) retracts after completion of the goal-directed task. The distance between the moveable platform upon which stimuli are placed and the edge of the table where the switch box is located is 40 cm. Once subjects initiate movement by lifting their hands off the switchbox, the platform retracts to a distance of 71 cm from the switchbox. **(C)** The physical geometry of the reverspective stimulus from a top view (solid lines) with respect to the target (red disk). The solid lines also represent the veridical percept for the reverspective stimulus. The dotted lines in **(C)** indicate its illusory percept. **(F)** illustrates the veridical percept and physical geometry of the proper-perspective stimulus. Note that the reverspective illusory percept in panel **(C)** matches the veridical percept of the proper-perspective stimulus in panel **(F)**.

To investigate how perceptual inputs affect underlying sensory-motor patterns, we ask participants to reach for a target located on a robust depth inversion illusion (DII). Visual illusions serve as the primary vehicle to test how skewed perceptual judgments of the environment may leak into our motor actions. DIIs elicit perceived depth reversal of scenes in which physically concave angles are perceived as convex and vice versa (Papathomas, 2007). The paradigm implemented in this study utilizes an actual physical set of stimuli built by an artist and an engineer to adequately fit the reaching workspace (in contrast to a set of stimuli presented on the computer screen). The set used for the study is part of a broad class of DIIs commonly referred to as reverspectives, in which the painted perspective cues compete with the physical geometry (Figure 3). The full presentation of the stimuli is described in detail elsewhere (Nguyen et al., 2014) but we briefly describe it here as well.

The reverspective stimulus is peculiar because it is a bistable stimulus. Generally, what we perceive visually depends on two main types of influences: (a) On one end are data-driven cues, commonly referred to as bottom-up cues, which are directly available to and processed by early stages of sensory mechanisms that are specifically tuned to those cues. Examples of such cues are edge orientation, direction and speed of motion, luminance and chromatic contrast, as well as binocular disparity (Papathomas and Bono, 2004; Rogers and Gyani, 2010; Keane et al., 2013). (b) On the other end, these cues are further processed and interpreted by what are commonly referred to as top-down influences (Rock, 1983; Wertheim, 1987; Gregory, 1997) such as endogenous attention, expectations and stored visual knowledge (either innate or acquired through experience); these influences can be formulated in a Bayesian framework (Langer and Bülthoff, 2001; Weiss et al., 2002; Kersten et al., 2004). Examples of such top-down influences are rule-based knowledge [e.g., that light comes from above (Kleffner and Ramachandran, 1992; Adams et al., 2004)], or that most objects are convex (Hill and Bruce, 1994; Langer and Bülthoff, 2001), as well as object-specific knowledge (e.g., that faces are convex overall, or that chairs generally have four legs).

In reverspective stimuli, these two types of influences compete against each other because they signal two different depth arrangements. Bottom-up cues, especially binocular disparity and motion parallax recover the true depth arrangement. On the other hand, prior experience with perspective (such as “a retinal trapezoid is actually a slanted rectangle” and “the edge of such a trapezoid with a larger retinal projection than the opposite edge is closer to me”) yields the opposite depth arrangement (Papathomas and Bono, 2004; Rogers and Gyani, 2010). Thus, the same stimulus gives rise to two vastly different percepts, veridical and illusory, enabling us to test how different percepts of the same stimulus affect sensory-motor behavior. In addition, the target was placed on a surface whose perceived spatial orientation under the illusion differed by nearly 90° from the physical orientation, thus greatly facilitating testing whether or not goal-directed movements are influenced by perceptual inputs. Furthermore, it is unknown how the moment-by-moment statistical variability inherent to transitional motor segments is affected by the reverspective.

We reason that by using the new experimental paradigm and statistical metrics, we may be able to determine how deliberate actions and supplemental motions are affected by the two different percepts of the reverspective stimulus. The key question is if the (empirically estimated rather than theoretically assumed) moment-by-moment motor fluctuations can separate the participants' perceptual states. We posit that how one perceives the reverspective may automatically pre-dispose one's hand orientation in anticipation of grasping the target. *We hypothesize* that if participants reached toward the target based on the illusory percept, the hand-path curvature would essentially change in some way as the hand translates and rotates in route to the target. Conversely, if reaching for the actual physical target, the hand would orient differently and change the path to avoid collision with the stimulus. It is the bistability of the DII that makes it possible to derive the illusory percept that evokes the arm-hand motion in the first place. Among the infinite number of ways to reach toward the target, if our hypothesis is correct, the two different percepts would unambiguously and systematically constraint the hand translational and rotational paths as a function of the illusory or veridical state.

Previous modeling (Torres and Zipser, 2002; Torres and Andersen, 2006) and experimental work on the motor psychophysical aspects of the pointing and reach-to-grasp family of movements across the personal workspace has informed us that the starting configuration of the reach determines its final configuration (Torres and Zipser, 2004; Soechting et al., 1995; Buneo et al., 1997; Desmurget and Prablanc, 1997; Desmurget et al., 1998). Thus the forward movements deliberately aimed to orient and translate the hand to match the perceived target are bound to constraint the initiation of the retracting motions and consequently impact the ensuing path back to rest, thus constraining the variability of the hand toward the resting position. However, since the presentation of the stimulus type randomly alternates, and these retracting movements are not instructed or following a visual goal, more variability is expected in the paths as the hand automatically goes back to rest. It is thus possible that these supplementary movements do not comply with previous results from motor psychophysics. In other words, the hand trajectories returning to rest may not reflect the mental states of the participant as evoked by the illusory or the veridical percepts, or to the same extent as reflected on the deliberate forward paths. If they did, then it may be of interest to include in future illusion experiments those goal-less movements that are now often discarded as a nuisance. Here we aim at characterizing their inherent statistical signatures to help further refine our inferences of mental states during intentional actions, as well as during rather automated movement segments that we are not entirely aware of.

### Participants

All methods and measurements presented in this study have been approved by the Rutgers IRB Committee in accordance with the Helsinki Act. A detailed visual explanation of the methods can be found in the Journal of Visualized Experiments (Nguyen et al., 2014). Sixteen participants were included in this study to assess the perceptual influences of the illusion on motor control. An additional set of 78 participants (including some of the 16 participants in the illusion task) performed a simple (baseline) point-to-a-dot task devoid of complex percepts. Using the inherent statistics of the full forward and back pointing loop in this set we empirically estimated the PDFs and moments of the distributions. This is in contrast to a priori assuming the Gaussian PDF and moments. We use the data in Figure 2 to illustrate the need to do this estimation more systematically in studies of action perception loops, as opposed to using a “one-size-fits-all” approach under assumed theoretical distribution parameters (as in Figure 1A).

The sixteen participants in the perceptual task of this work were screened for visual acuity, stereopsis using a Random-Dot Stereo Test, and eye dominance. Written informed consent of the Rutgers University Institutional Review Board approved protocol in compliance with the Declaration of Helsinki was obtained before beginning experimental sessions. Compensation was provided for partaking in each experimental session. We recruited healthy right-handed participants with normal or corrected-to-normal visual acuity. Exclusion criteria for participants are as follows: (1) if participants experienced difficulty in perceiving the illusion and the blurring lens used to reduce stereopsis caused discomfort or dizziness; (2) if participants had existing drug and/or alcohol dependencies as it is known that binocular depth inversion is impaired in these populations (Schneider et al., 1996; Leweke et al., 1999); (3) if only partial data was obtained due to malfunctioning of the motion-capture system or excess sensitivity to electromagnetic fields, causing significant losses in data. The Motion Monitor software (The Motion Monitor, Innovative Sports Training, Inc., Chicago, IL) was used to build metal maps and assess prior to the experiments the stability of the electromagnetic field so as to eliminate any disturbances. However, prior to analyses the data was always pre-processed all data to screen for any distortions that the first procedure did not catch.

The 16 participants were eight males (ages 21–35 years old) and eight females (ages 19–33 years old). All subjects were right handed. We selected a sample size of *N* = 16 since comparable studies used a similar number of subjects (Hartung et al., 2005; Króliczak et al., 2006; Wagner et al., 2009; Prime and Marotta, 2013).

### Motion capture

We used 15 electromagnetic sensors at a sampling frequency of 240 Hz (Polhemus, Liberty, Colchester, VT) and motion-tracking software (The Motion Monitor, Innovative Sports Training, Inc., Chicago, IL) for continuous motion capture. Sensors 1–12 were placed on the following body segments using sports bands designed to optimize unrestricted movement of the body: center of the forehead, the trunk at thoracic vertebra T12, right and left scapula, left upper arm, left forearm, left wrist, right upper arm, right forearm, right wrist, right hand index finger, and right hand thumb (Figure 3A). An additional sensor was used to digitize the body to construct a biomechanical model using the Motion Monitor software. The remaining two sensors were placed on the backside of each stimulus directly behind each target's location to attain an accurate position of each target in 3D space relative to the participant during the training and experimental blocks. We recorded the full motor response of each participant in real-time both in the forward motion (from initiation of hand movement up to their furthest reach), and in the non-instructed, automatic retraction of the arm back to resting position. This paper focuses on the hand data.

### Stimuli and apparatus

In order to present physical stimuli to the participant, a moveable platform on a sliding track was constructed on a table at an appropriate height that allowed for the stimulus to be presented at eye-level when the participant was seated in front of the table. A spring mechanism was used to control the retraction of the stimulus platform. A set of lamps placed behind the participant's chair illuminated the stimulus platform evenly (see below description of the computerized control system for the lights and switch box), since uneven lighting may cast shadows that interfere with perceptual judgments. A switch box was added to the edge of the table closest to where the participant was seated. Participants placed their right hand on the switch box at the beginning of each trial and activated the switch as soon as they lifted their hand to execute the reach movement (Figures 3C,F).

The switch box, lights, and spring mechanism for the stimulus platform were connected to a microcontroller (Arduino, Smart Projects, Italy) that executed the simultaneous activation of the retraction of the stimulus platform via the spring mechanism and the turning off of lights once the switch box is triggered. The stimulus must retract and the lights must turn off after the initiation of the reach movement in each trial to prevent any online visual corrections and haptic feedback from occurring. The switch box mechanism was used so that retraction of the stimulus and the onset of darkness were implemented only after the movement had been initiated to produce an immediate reach task. This is a critical detail to emphasize, as researchers argue that a delay in movement onset after the removal of visual and haptic cues results in a memory-guided reach, relying on ventral stream contributions, whereas real-time motor planning only occurs when visual information is present at the time of movement onset (Westwood and Goodale, 2003; Prime and Marotta, 2013). By employing an immediate reach task, our methods address possible influences of top-down signals from the ventral visual stream on automatic motor strategies upon approach of the target on the DII stimulus.

A set of training stimuli was utilized to familiarize participants with the paradigm prior to exposure to the test stimuli (Nguyen et al., 2014). Training stimuli consisted of two painted rectangular panels representing the isolated right surface wall of the middle building embedded in the reverspective stimulus and the proper-perspective stimulus. Each rectangular panel assumed the same spatial orientations as that of the right-hand side wall of the middle building found in the 3D scene for both the proper- and the reverse- perspective stimuli (Figure 3). Participants were able to recover the true physical slant of the training stimuli at all times. Red planar disk targets were positioned on the training stimuli to match the location of the elliptical red planar disk targets on the experimental stimuli.

To determine whether or not top-down visual processes affect sensory-motor control, two 3D stimuli, a proper-perspective and a reverspective (the DII), were used to elicit three distinct experimental conditions (Figure 3). For the proper-perspective (Figures 3D–F), the perspective-painted cues were congruent with the bottom-up signals of binocular disparity and motion parallax elicited by the physical geometry of the stimulus. This congruency produces a stable concave percept of two concave truncated pyramids with two streets that recede away from the viewer: the right wall of the central building slants with its right vertical edge further away than the central vertical edge. For the reverspective (Figures 3A–C), the painted cues compete with bottom-up signals that are elicited by the physical stimulus, thus creating a bistable stimulus that produces two main percepts: (a) a veridical depth configuration of two convex truncated pyramids that protrude toward the viewer, in which the right wall of the central building slants with its right vertical edge closer to the viewer than the central vertical edge (indicated by solid lines in Figure 3C), and (b) an illusory inverted-depth configuration (indicated by dotted lines in Figure 3C) of two concave truncated pyramids with two streets that recede away from the viewer, similar to the percept elicited by the proper perspective. As a result of the illusory percept, the perceived 3D-orientation of surfaces is affected drastically. Specifically, the right wall of the central building is misperceived as slanted with its right vertical edge further away than the central vertical edge. Notice that the *perceived* spatial orientation of this wall is almost orthogonal to its *physical* orientation. The illusory depth inversion causes convexities and concavities to be perceived as concavities and convexities, respectively.

Elliptical red planar disk targets were placed on the proper- and reverse- perspective stimuli as shown in Figure 3. The perceived location and spatial orientation of the target on the proper perspective remains stable since the perceived depth configuration is static. In contrast, the perceived location and spatial orientation of the target in the reverspective depends on whether viewers obtain the illusion (in which case the perceived orientation will be nearly orthogonal to the true physical orientation), or do not obtain the DII (in which case its orientation will be veridical).

### Experimental protocol

All stimuli were placed out of view from the participant before starting the experiment. All lights were turned off, except for the lamps used to illuminate the stimulus platform. Any computer screens that were in use to run the experiment were dimmed so that their luminosities did not interfere with the even lighting projected onto the apparatus. Before beginning any trials, each participant was informed of the experimental procedure. The experimenter demonstrated how to grab at where he or she last viewed the target by approaching it perpendicular to the surface it was perceived on.

Three practice trials were first executed to allow the participant to become comfortable with the setup. Stimuli were not added to the platform at this time—only a black board with a center pole protrusion used to later attach training stimuli was visible. Participants reached toward the center pole and brought their hands back to rest upon completing the reach at their own pace. Note that no instructions were given on how to retract the hand; this component was unprompted and occurred automatically, largely below the subject's awareness.

After the practice trials, training trials were run on the training stimuli. The participant was instructed to close his/her eyes after each trial for the remainder of the experiment. The experimenter confirmed that the participant kept his/her eyes closed after each trial before affixing the next stimulus. The order of training stimulus presentation was randomized by the MATLAB program. Training stimuli help demonstrate the curvature of the reach when asked to grab at targets on physical surfaces representative of the targets used in the experimental stimuli (see Nguyen et al., 2014 for more details). There were four trials per stimulus, for a total of eight training trials.

Once training was completed, experimental trials were executed. Three stimulus conditions were used for the experimental trials: (1) reverspective under illusory percept (illusory), (2) reverspective under veridical percept (veridical), and (3) proper-perspective (proper). Recall that stimulus conditions 1 (illusory) and 2 (veridical) utilize the same physical reverspective stimulus.

The reverspective stimulus was presented first to determine if participants could stabilize the illusory percept of the middle building “popping out” toward them. If participants had trouble stabilizing the illusory percept, a de-focusing lens on the non-dominant eye was used to weaken stereopsis in order to preserve the illusory percept while maintaining reaching distance to the target. This method was employed to preserve binocular viewing conditions in previous work (Króliczak et al., 2006). If participants required the de-focusing lens, they were instructed to put them on before each illusory trial. There were a total of eight participants who required the de-focusing lens. There were no group differences between subjects that required a defocusing lens vs. those that did not. After the first illusory trial, the order of presentation of each stimulus was randomized. To ensure the presence of a stable percept for each trial, the experimenter, depending on the stimulus condition, gave the following instructions:

Illusory and Proper: “View the middle building as popping out toward you.” Veridical: “View the middle building as caving in away from you.”

Once the participant confirmed a stable percept, they were instructed to grab at the target, similarly to how they reached during training trials. Participants were not instructed to return to any specific position after each reach was completed, allowing for a natural, uninstructed retraction of the hand. Twelve trials for each condition were performed for a total of 36 experimental trials. All trials were performed under binocular viewing conditions. The choice of 10 trials per condition was sufficient to estimate the statistical parameters with high confidence, as these yielded over 100 measurements of angular velocity peaks (see below), the parameter of interest in the analyses. The additional two trials were in case of sensor malfunctioning and to ensure at least 100 peaks for the estimation procedure below.

### Data analysis

In order to address whether or not reaches performed on the reverse perspective stimulus under the illusory percept were similar to reaches made under the veridical percept on the same stimulus, entire trajectory paths starting from the initiation of movement to the return to resting position were analyzed in 3D space. We analyzed both the forward goal-directed reaches and the (uninstructed) supplementary, transitional movements to retract to a resting position. Each trial's trajectory was decomposed into two movement classes (forward, goal-directed reach and retracting, supplementary motions) by detecting the point at which the velocity of the movement, after its initiation, nears instantaneous zero velocity and the distance to the target location is near zero as well (Figures 2B,C).

We recorded 12 trials per condition and included those without any partial data loss. Data analyses included 11 trials per condition for each subject due to data loss in some participants. For participants that performed 12 trials per condition without any data collection errors, 11 out of 12 trials were randomly selected for analysis. Furthermore, we used kinematic parameters such as the angular velocity, with high frequency of peaks. This ensured that across trials, each participant had at least 100 measurements (peaks) for each condition. We can then estimate with high confidence the statistical parameters that empirically characterized the probability distribution family most likely associated with the random process of speed-dependent parameters underlying each condition.

### Raw kinematics

For the purpose of this original research article, we present data collected from the three sensors located on the dominant hand. Data analyses on the remaining nine sensors is beyond the scope of this paper (results will be disseminated in future work).

We test for significant differences of the positional hand paths using the Wilks' test statistic (Wagner et al., 2009) (p.180) for the paths' points individually. Hand path trajectories were position- and time-normalized for each participant. For each condition, all points in each trajectory family (i.e., family of curves for each percept) were translated to center the mean starting position at the origin. This was performed in order to account for variations in hand placement on the switch box that occurred across participants. Each forward and retraction trajectory is analyzed independently.

The data is divided into *k* groups, *k* = 3, corresponding to veridical, proper and illusion. Each group has sample size *n* = 11 × 16 sample paths, from *16* subjects × *11* repetitions per condition. For each condition, trajectory paths were resampled to 100 points. This unit speed treatment of the curve ensured that each point along the geometric path for each trial was equally spaced from the previous point. This is necessary in order to calculate the Wilk's lambda test statistic of the geometric curve [see for example (Torres and Zipser, 2002, 2004; Torres and Andersen, 2006) for similar applications of the Wilk's lambda test statistic to kinematics data and also for justification of the procedure, as in the primate hand-arm-movements space and time are separable (Atkeson and Hollerbach, 1985; Nishikawa et al., 1999)].

We compare the mean vectors of the *k* samples searching for significant differences and pose the null hypothesis:

$$\begin{array}{l} H_{0}:\mu_{Proper}=\mu_{Veridical}=\mu_{Illusion}\text{ vs}.\;H_{1}\text{ at least two }\mu \text{ are unequal}. \end{array}$$

To test the null hypothesis we compare the paths of each condition using the Wilk's lambda test, a multivariate generalization of the univariate F-distribution, which allows for the reduction of the likelihood test statistic Λ to a scalar value by way of determinants (Rencher and Christensen, 2012).

Since each three-dimensional vector is analyzed along the hand trajectory, the Wilk's lambda test statistic helps deduce whether or not a pairwise difference of mean vectors is significant. Critical to this step is that the vectors span a trivariate normal distribution which we test below through error simulation and graphical visualization.

The Wilk's lambda statistic uses the likelihood ratio test $\Lambda =\frac{\text{det}\left(E\right)}{\text{det}\left(E+H\right)}$, in which the “within” sum of squares and products are matrix E, and the “total” sum of squares and products is matrix (E+H). The matrix $E=\underset{ij}{\sum }y_{ij}y_{ij}^{t}-\overset{k}{\underset{i}{\sum }}\frac{1}{n}y_{i.}y_{i.}^{t}$, where *y*_*ij*_ is a sample point and $y_{i.}=\overset{n}{\underset{j}{\sum }}y_{ij}$ is the total sum of the *ith* sample. The matrix $H=\overset{k}{\sum }\frac{1}{n}y_{i.}y_{i.}^{t}-\;\frac{1}{kn}y_{..}y_{..}^{t}$, in which $y_{..}=\overset{k}{\underset{i}{\sum }}\overset{n}{\underset{j}{\sum }}y_{ij}$ is the overall total.

When Λ ≤ Λ^*^_∝, *p, vH, vE*_, (Λ is small) the null hypothesis is rejected. In Λ^*^_∝, *p, vE, vH*_, ∝ is the level of confidence, *p* is the number of variables or dimensions (3 dimensions), *vH* = *k*−1 and *vE* = *k* (*n*−1) are the degrees of freedom for hypothesis and error respectively, where *k* is the number of conditions (3, Proper, Illusion and Veridical) and n is the number of trials (*11* repeats × *16* subjects).

For our experimental design, the confidence interval ∝ = 0.05, *vH* = (3−1) = 2 since the number of samples is *k* = 3 for three conditions, and *vE* = 2(11^*^16−1) = 350 since 11 trials per condition were analyzed for each of the 16 participants (*n* = 11^*^16 = 176). The critical values of Λ^*^_∝, *p, vH, vE*_ used to determine statistical significance are _Λ^*^_lower_ = Λ^*^∝ = 0.05, *p* = 3, *vE* = 320, *vH* = 2_ = 0.961 and _Λ^*^_upper_ = Λ^*^∝ = 0.05, *p* = 3, *vE* = 440, *vH* = 2_ = 0.972, as taken from Rencher & Christensen Table A.9.

These values define the range in which Λ^*^_∝ = 0.05, *p* = 3, *vE* = 350, *vH* = 2_ lies. Therefore, values of Λ > [Λ^*^_lower_:Λ^*^_upper_] cannot reject the null hypothesis (This boundaries are represented in **Figure 7** by a dashed line). For each point in the path we perform this test at the 0.05 level of confidence. These are 100 tests per comparison (three comparisons, Veridical vs. Illusory; Veridical vs. Proper; Illusory vs. Proper) for a total of 300 comparisons yielding 300 Λ values. Of each of the the 100 Λ values in a path-path comparison we take 25% segments of the path to further test the distribution of Λ values as the movements unfold forward and as they retract. All points in each 1/4 of the path are measured against the critical value to examine the null in each segment and to determine which part of the path is the strongest rejecting the null. To visualize the Λ values we use conventional box plots. On each box, the central mark is the median, the edges of the box are the 25th and 75th percentiles, the whiskers extend to the most extreme data points not considered outliers, and outliers are plotted individually. For example, the boxplots in Figures **7A–C** divide the path into four segments, each with 25 values of Λ used in the drawing of the box plots. The figure marks the critical value with a dashed thick line. Any values below this dashed line reject the null at the 0.05 alpha level. Those above the line cannot reject the null (e.g., **Figure 7C** comparing the path geometry of the Illusory and Proper percepts).

To ensure that we properly estimate confidence regions from the errors using the trivariate normal distribution (see below) as well as to provide graphical representation of the data in terms of the 100(1-α)%-three dimensional confidence region around the mean path for the trivariate case (Rencher, 1995, p.180) we use: $\left\{\frac{\upsilon -p+1}{\upsilon p}a^{t}{\text{S}}^{-1}a<F_{p,\upsilon -p+1,\alpha }\right\}$ with degrees of freedom υ = *n*−1, *p* = 3, α = 0.05 to estimate at that level of confidence, and $\text{S}=\frac{E}{nk-k}$ (Rencher, 1995) as an empirical estimate of the sample variance-covariance matrix Σ = *E*[*S*], *E*[.] is the expected value operator, *n* is the number of trials as before (11 trials × 16 subjects) and as before, *k* the number of conditions [3 (percept types) × 2 (forward, retraction)], *a* is the point. The term *na*^*t*^***S***^−1^*a* is the **T**^2^ statistic test converted to an **F**-test (Rencher, 1998; Theorem 3.3.B p.67).

We generate random vectors around each point in each path to simulate errors in a normal trivariate distribution. The radius around the estimated mean is chosen as the maximum distance from all the points in the sample to the sample mean ±ϵ close to (0,0,0). We then evaluate the inequation above and reject the values above the *F*_3, 176, 0.05_ value for each forward and retraction family of curves. The maximum of the values we accept is set as the “at least” 95% confidence region around each mean point in the path. To build the mean path for each sample group *k*, we estimate the mean point in the paths across the sample (*11* × *16* for each of the *100* points). The 95%-confidence region surface surrounding each thus estimated mean path is drown by plotting a circle of radius equal to the corresponding estimated confidence region value around each empirically estimated mean path point, and connecting each of these circles along the path. For example, visualization of the three-dimensional confidence regions and the estimated mean paths around them are shown in **Figure 6A** for the forward case and **Figure 6B** for the retractions.

### Changes in hand orientation toward the target

To quantify possible differences in hand orientation toward the target, positional information from sensors located on the thumb, index finger, and wrist of the right hand at the end of the forward goal-directed reach was analyzed. Since perceived veridical and illusory surface orientations on the reverspective stimulus differ by nearly 90°, the angle at which the hand approached the target may show an effect if the illusory percept impacted the immediate reach. Alternatively, because of the abundant DoF in the arm-hand linkage, the system could lock some DoF at the end effector and maintain similar hand orientations across conditions, by rather allowing the variability along the dimensions of other joints in the upper extremities. In other words, the bi-stable illusion could produce different endpoint paths traversed with similar hand orientations.

We compare orientation related quantities defined below for significant differences and as before we pose the null hypothesis:

$$\begin{array}{l} H_{0}:\mu_{Proper}=\mu_{Veridical}=\mu_{Illusion}\text{ vs}.\;H_{1}\text{ at least two }\mu \text{ are unequal}. \end{array}$$

**Figure 4:**
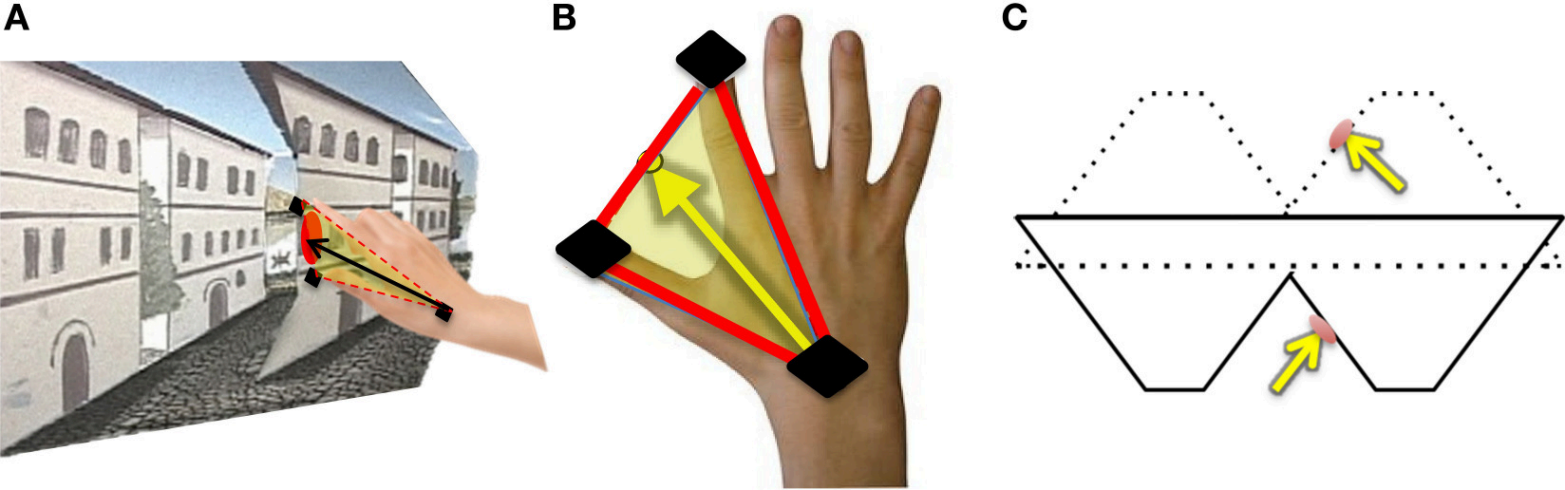
**Hand orientation analytical methods: (A) The participant reaches toward the target on the reverspective stimulus**. **(B)** Illustration of how hand-approach unit vectors are calculated using sensors located on the thumb, index finger, and wrist (black). The midpoint between the thumb and index finger is used to define the vector of approach with respect to the wrist sensor position. **(C)** The unit vector normal to the target location on the reverspective stimulus (solid lines) and the proper-perspective stimulus (dotted lines).

For each trial, normalized approach unit vectors were derived by calculating the midpoint between the thumb and index finger in relation to the wrist position for each trial (Figures 4A,B). We defined all possible angle configurations when comparing approach unit vectors in each condition for every participant by taking the dot product for each unit approach vectors group. The dot product is given by ***A* · *B* = ‖ *A* ‖‖ *B* ‖ *cos*θ**, in which **‖ *A* ‖** and **‖ *B* ‖** represent the magnitudes of approach unit vectors, and **θ** represents the angle between *A* and *B*. Denoting *Î* for illusory, $\hat{P}$ for proper and $\hat{V}$ for veridical, for each participant, $\left[{\hat{\text{I}}}_{1,\ldots 11}\right]\cdot \left[{\hat{\text{P}}}_{1,\ldots 11}\right]$, $\left[{\hat{\text{I}}}_{1,\ldots 11}\right]\cdot \left[{\hat{\text{V}}}_{1,\ldots 11}\right]$, and $\left[{\hat{\text{V}}}_{1,\ldots 11}\right]\cdot \left[{\hat{\text{P}}}_{1,\ldots 11}\right]$ is calculated, resulting in 121 angle values per comparison per subject. Since the frequency histograms of these motion parameters are non-symmetric, the non-parametric ANOVA, the Kruskal-Wallis Test, is utilized to determine whether or not each angle comparison group (∠ Illusory vs. Proper, ∠ Illusory vs. Veridical, and ∠ Veridical vs. Proper) is statistically different from one another (Ross, 2009). The Kruskal-Wallis Test determines if the mean ranks from each condition are similar. The null hypothesis of the Kruskal-Wallis Test indicates that each condition comes from the same distribution. The alternative is that at least two conditions come from different distributions.

In addition to finding all possible angle configurations between each approach unit vector, the mean approach unit vector for each condition was determined to compute the angles formed with the unit vector normal to each stimulus' target location (Figure 4C). Each possible angle is calculated using the dot product, as previously discussed, to obtain $\left[{\hat{\text{I}}}_{1,\ldots 11}\right]\cdot \left[{\hat{{\hat{\text{T}}}_{\text{R}}}}_{1,\ldots 11}\right]$, $\left[{\hat{\text{V}}}_{1,\ldots 11}\right]\cdot \left[{\hat{{\hat{\text{T}}}_{\text{R}}}}_{1,\ldots 11}\right]$, and $\left[{\hat{\text{P}}}_{1,\ldots 11}\right]\cdot \left[{\hat{{\hat{\text{T}}}_{\text{P}}}}_{1,\ldots 11}\right]$, in which ${\hat{\text{T}}}_{\text{R}}$ and ${\hat{\text{T}}}_{\text{P}}$ represent the target surface unit vector normal to the reverspective stimulus and the proper-perspective stimulus, respectively. To further elucidate the relationship between hand orientations and target locations on each stimulus, hand approach unit vectors for each condition were also compared to the target surface normal of the proper-perspective for the illusory and veridical mean approach unit vectors ($\left[{\hat{\text{I}}}_{1,\ldots 11}\right]\cdot \left[{\hat{{\hat{\text{T}}}_{\text{P}}}}_{1,\ldots 11}\right]$, $\left[{\hat{\text{V}}}_{1,\ldots 11}\right]\cdot \left[{\hat{{\hat{\text{T}}}_{\text{P}}}}_{1,\ldots 11}\right]$), and the reverspective for the proper mean approach unit vector $\left(\left[{\hat{\text{P}}}_{1,\ldots 11}\right]\cdot \left[{\hat{{\hat{\text{T}}}_{\text{R}}}}_{1,\ldots 11}\right]\right)$. This allows for us to identify whether or not there are similarities in hand approach under the illusory, veridical, and proper conditions.

### Speed profiles

To investigate whether or not changes in speed profiles occur for the task required in each condition, motor output fluctuations in performance from the trial-by-trial in the angular velocities of each trajectory family were analyzed. By a trajectory family we mean those curves with a statistically separable geometric shape corresponding to each perceptual condition (veridical, proper, or illusory).

**Figure 5:**
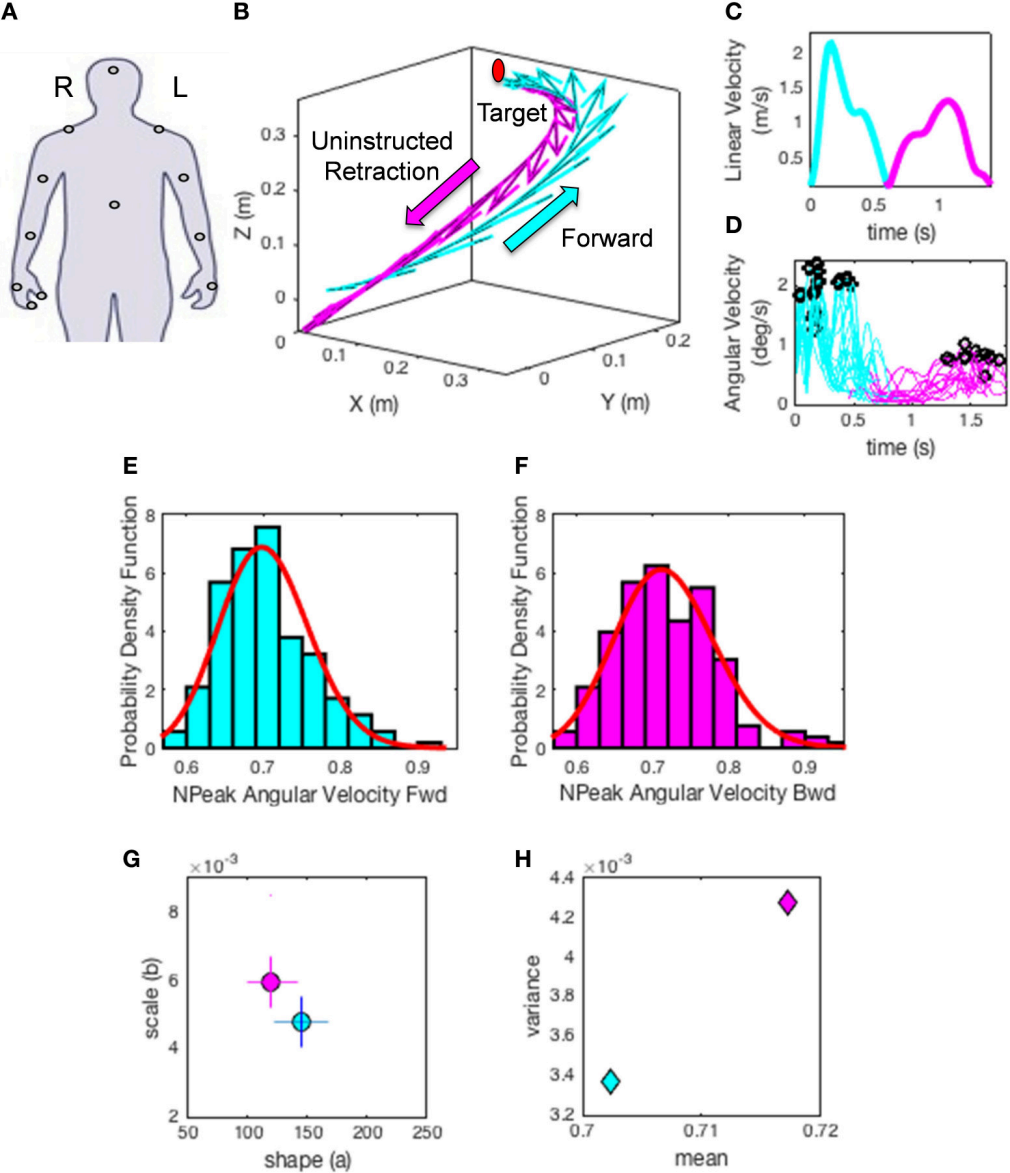
**Methods illustration**. **(A)** Location of each sensor as affixed on the participant's limbs. XYZ coordinates (0, 0, 0) indicate the starting position of the hand on the switchbox. Moving in the positive Y direction indicates the forward motion toward the target, whereas the x- and z- axes are for the horizontal and vertical dimensions, respectively. **(B)** A sample hand trajectory for a participant in the forward (cyan) goal-directed reach and the uninstructed retraction back to rest (magenta). For illustration purposes the arrows indicate a down-sampled set of velocity vectors throughout the trajectory profile (the sampling resolution of 240 Hz gives a very large number of such vectors, all of which we use in the analyses). The initial target location is shown in red to demonstrate where participants were reaching. The movement trajectory is split into two components by finding the point at which the linear hand speed nears instantaneous zero velocity **(C)**. The magnitude of each sampled velocity vector from the motion trajectory is obtained using the Euclidean norm. This yields a speed profile over the motion time in **(C)**. Underlying the linear displacements is the peak angular velocity registered for each joint and for each movement class. A subset of angular velocity measurements are shown in **(D)** corresponding to the linear speed of **(C)**. The peak angular velocities are given by the black points along the angular velocity traces. The peak angular velocity is normalized (see text). The underlying probability distribution for the normalized peak angular velocity profiles **(E,F)** are empirically estimated using the continuous Gamma family of probability distributions (red curve) for each movement class. **(G)** shows the Gamma parameter plane with the estimated shape (*a*) and scale (*b*) parameters using maximum likelihood estimation. The 95% confidence intervals are also plotted for the given example. Points on this map provide a characterization of how external perceptual stimuli affect the fluctuations of the speed of trajectories in each condition, thus making it variable from trial to trial. Note the differences in the shape and scale parameters of the forward reach (cyan) and uninstructed retraction (magenta) in **(G)**. **(H)** The estimated moments, means (μ_*W*_) and variances ($\sigma_{W}^{2}$) for the given trajectories using the empirically estimated shape (*a*) and scale (*b*) parameters. This plot provides an additional metric to characterize the influence of perceptual changes on sensory-motor control.

The following steps explain the analytical methods:

(1) The hand trajectories are harnessed for each condition and movement class. Figure 5A shows the hand locations where sensors were placed (e.g., right dominant hand). Figure 5B shows the trajectories with the tangential velocity vector flows forward and back. Figure 5C shows the linear speed obtained from the velocity trajectories. Figure 5D shows the underlying angular speed profiles reflecting the changes in hand orientation along the trajectory to match the target. The colors represent the movement type.
(2) The peak angular velocities are harnessed and normalized to bring all fluctuations to a similar scale and avoid confounds due to anatomical differences across subjects (Lleonart et al., 2000). To this end each peak between two local minima are obtained. The peak angular velocity value is divided by the sum of the peak and the average angular velocity between the two minima.
  $$\begin{array}{l} nPVindex=\frac{PV}{PV+Average\left(V_{\min\text{ to }\min}\right)} \end{array}$$
  This yields the normalized peak angular velocity index. This is our parameter of interest and its time-series is the signal of our interest to empirically estimate the PDFs. Note here that higher values of this index indicate lower speed on average.
(3) Plot the frequency histograms (Figures 5E,F) of the parameter using optimal binning (Freedman and Diaconis, 1981; Shimazaki and Shinomoto, 2007) and estimate the underlying family of probability distributions best characterizing the trial-to-trial fluctuations in performance of the index above (other kinematics parameters can also be used). Besides individual estimation, this procedure can also be done for the ensemble data. Here the normalized angular velocity indexes are grouped by condition for the forward goal-directed reach and the retraction movement across subjects. Each histogram comprises at least 100 measurements per person.
(4) Using maximum likelihood estimation we empirically obtain from the data the values and ranges of the shape (*a*) and scale (*b*) parameters of the continuous Gamma family of probability distributions. The Gamma PDF is given by:
  $$\begin{array}{l} y=f\left(x\;|\;a,b\right)=\frac{1}{b^{a}\Gamma \left(a\right)}x^{a-1}e^{\frac{-x}{b}} \end{array}$$
  in which *a* is the shape parameter, *b* is the scale parameter, and Γ is the Gamma function (Ross, 1996). We then plot the estimated Gamma-parameters for each participant with 95% confidence intervals on the (*a, b*)-Gamma parameter plane. In this way we localize the individual participant and compare each subject's location to those of the other subjects (Figure 5G). Here we also look at the ensemble data to identify self-emerging clusters and patterns. In this context we do so in relation to different perceptual conditions.
(5) Obtain the moments of the estimated distribution using the estimated Gamma-parameters. Figure 5H shows the first moment (the mean) and the second moment (the variance) localizing the subject on the parameter plane for the two representative movement types. Then using the estimated moments obtain the noise to signal ratio [the Fano Factor (Fano, 1947) FF = empirically estimated Gamma variance/empirically estimated Gamma mean]. The Gamma mean is given by μ = a^*^b and the Gamma variance is given by σ = a^*^b^2^. Notice that the noise-to-signal ratio, the Fano Factor in this case, is also the Gamma scale parameter $b=\frac{\sigma^{2}}{\mu }=\frac{a.b^{2}}{a.b}$ (Ross, 1996).

This is important as we are assessing the levels of noise in relation to the empirical estimation of the Gamma parameters from the data as a function of percept type. Higher levels of noise will correspond to increases of the *b* ***scale*** parameter along the vertical axes of the Gamma plane; whereas lower levels of noise will correspond to lower values along the scale axis of the Gamma plane.

It is also important to emphasize that when the ***shape*** parameter *a* of the Gamma family *a = 1* the data follows the *memoryless* Exponential probability distribution, a special case in the Gamma family. This is the most random distribution whereby events in the past do not accumulate information predictive of events in the future (Ross, 1996). Larger values toward the right of the shape axes on the Gamma (*a, b*)-plane tend toward the symmetric distributions, with a variety of skewed distributions in between the two extremes (Ross, 2014).

In the text we will refer to the level of randomness by examining the value of the empirically estimated shape parameter. When increasing the shape value to the right of the horizontal axis, we will refer to the accumulation of information toward the Gaussian range of the Gamma parameter plane. Likewise we will refer to higher or lower noise levels according to the empirically estimated *b* Gamma-scale parameter value, (the FF).

The Gamma distribution family has been previously used to empirically parameterize the variability inherently present in natural human motions, ranging from normal (Torres, 2011, 2013a) to pathological (Torres, 2012; Torres et al., 2013, 2014). For example, Figure 2 is used here to illustrate the evolution of the empirically estimated parameters in a cross-section of the typical human population across different developmental stages and typical aging stages. This map was determined using the normalized linear velocity peak index (as described above for the angular velocity case) from full loop pointing behavior in a simpler task without the perceptual stimuli, but containing similar biomechanics' structure as the task used here. The shifts in stochastic signatures quantified in Figure 2 from young to mid-age adulthood illustrates the need to perform this estimation individually as the average speed and the degree of skewness, kurtoses, and dispersion change with typical development, maturation and aging.

Within the typical ranges of the human population, our Gamma-distribution based empirical estimation should therefore characterize potential differences between the types of movements generated by each stimulus. If the reverspective illusory condition instigated a trajectory speed profile similar to that of the proper-perspective condition, then their Gamma distributions are hypothesized to be similar despite random presentations and associated inherent variations. In contrast, if it is similar to the reverspective veridical condition, then both of these reverspective conditions should reveal similar Gamma distributions for normalized peak angular velocity values. Because of the complexity of the task we use fewer trials than 100. For statistical power and robust estimation with tight confidence bounds we rely instead in the higher frequency of peaks in the angular speed. This is why with a minimum of 10 trials per person we can gather enough measurements (over 100) for our estimation. This is in contrast for example with Figure 2 where we use the linear velocity peaks as the parameter of interest for the estimation and a minimum of 100 trials per person.

We underscore that the empirical estimation of the appropriate family of probability distributions to characterize sensory-motor control stands in contrast to traditional approaches assessing motor variability. The latter simply assumes a theoretical distribution (e.g., the Gaussian) and takes the (assumed) mean and variance of kinematic parameters linked to the motion trajectories across a number of trials (as in Figure 1A). The probability distributions empirically derived from unconstrained movements in three dimensions are in fact skewed, governed by power laws and inclusive of multiplicative noise (Torres, 2011, 2013a).

The recent work has shown how these parameters change as sensory-motor behavior adapts to external stimuli, providing human motion parameter range for the values of the Gamma shape ***a***. Pathological cases fall at or near ***a*** = *1* (Torres et al., 2010, 2013; Torres, 2012), the special Exponential case of the Gamma distribution. In contrast skilled athletes with high certainty in the predictive value of the speed in their impending trajectories have symmetric, Gaussian-shaped distributions of their speed-dependent parameters (Torres, 2011, 2013a). The recent work set bounds on these parameters that will help us interpret results in the context of this new, illusion-driven, visuomotor task.

## Results

### Differences in the unfolding of movement: hand path trajectory analyses

**Figure 6:**
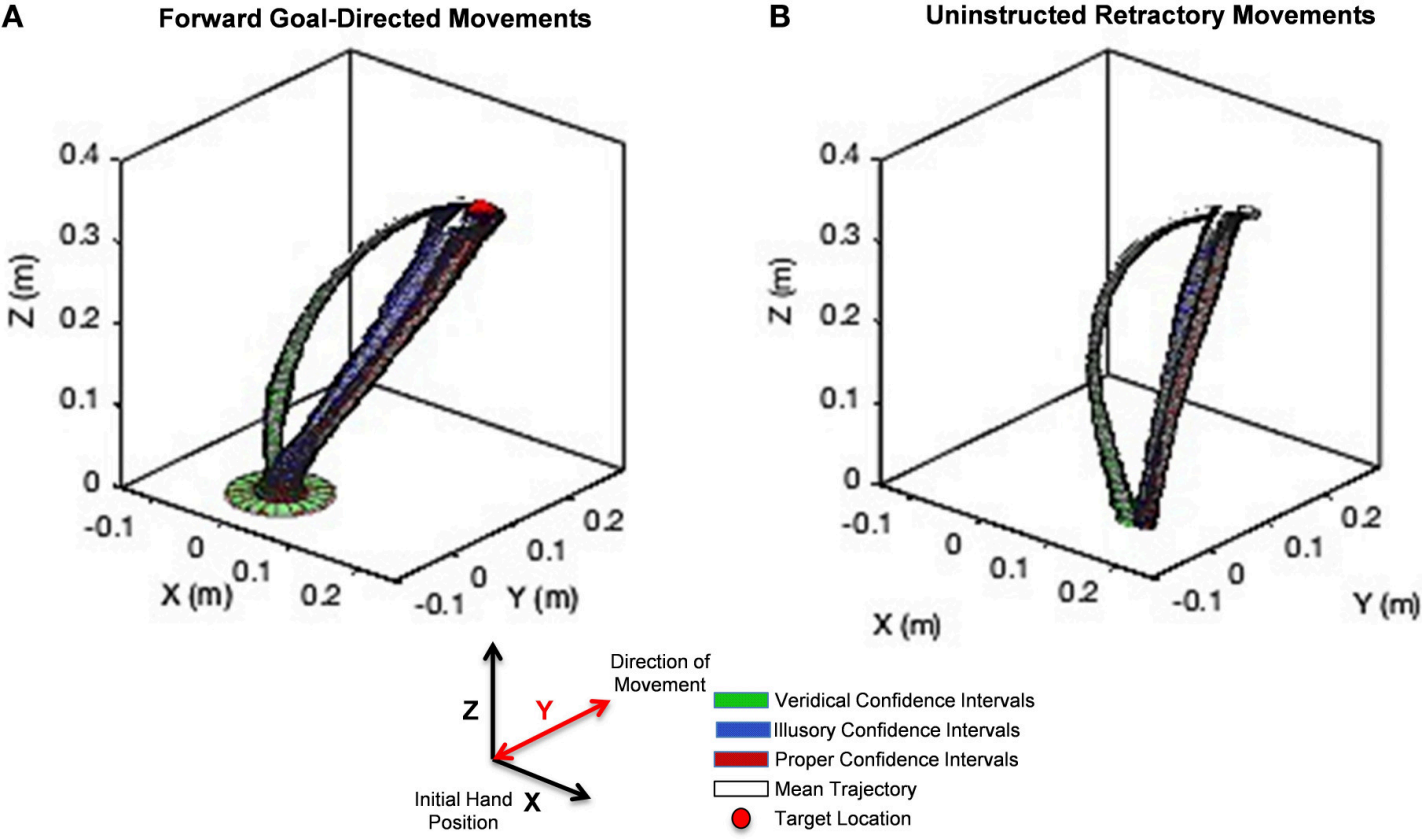
**Unfolding statistics of movement trajectories to Target and back to rest**. **(A)** The hand path trajectory for the forward goal-directed movement. **(B)** Results for the uninstructed retraction of the hand back to rest. Hand path trajectory confidence intervals under the veridical percept are illustrated in green, under the illusory percept in blue, and under the proper percept in red. The mean of each trajectory family is designated in white. A red sphere in **(A)** denotes the location of the target on the stimulus. The y-axis denotes the direction of movement toward the target and back to rest. Note the curvature of reach under the veridical percept (green) is markedly different from those performed under the illusory percept (blue), although both conditions share the same physical stimulus. The illusory percept hand path trajectory follows a similar path found in the proper condition (red). This behavior is maintained not only in the forward, goal-directed movement, but even more so in the uninstructed retraction of the hand.

**Figure 7:**
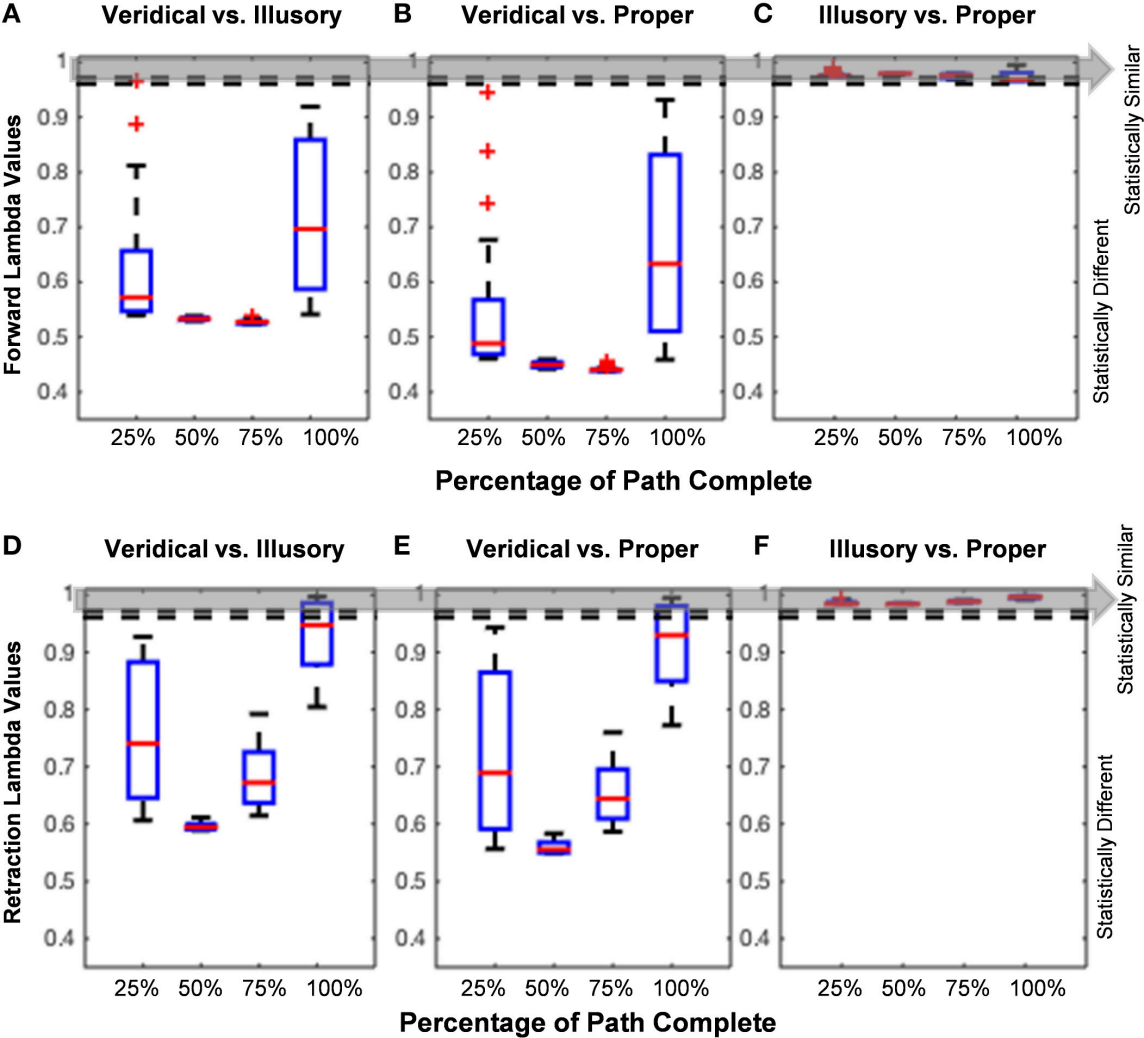
**Hand path trajectory analysis**. **(A–C)** and **(D–F)** show pairwise comparisons of each condition's hand path trajectory using calculated Wilk's Lambda Values. Red lines found within each boxplot indicate the median Lambda value. The horizontal edges of each blue boxplot represent the 25th and 75th percentiles. Thick black dotted lines on each end of the boxplot indicate values that are not considered outliers, and red plus signs indicate any outlier values, such as in Figures 5A,B. Since the unfolding of movement is critical in determining how a participant performs each trial, the Lambda Values are calculated based on percentage of path completed (e.g., 25, 50, 75, and 100%). The Wilk's Lambda Test Statistic is given by $\Lambda {*}_{lower}=\Lambda {*}_{\propto \;=\;0.05,p\;=\;3,vE\;=\;320,vH\;=\;2}=0.961$ and $\Lambda {*}_{upper}=\Lambda {*}_{\propto \;=\;0.05,p\;=\;3,vE\;=\;440,vH\;=\;2}=0.972$, given by the dotted lines and gray area. If $\Lambda \leq [\Lambda {*}_{lower}:\Lambda {*}_{upper}]$ (below the dotted lines and gray area), the trajectory families are statistically different. **(A,D)** show that veridical and illusory hand path trajectories are statistically different from one another in both the goal-directed movement **(A)** and uninstructed retraction **(D)**. The same behavior is observed in the comparison of the veridical vs. proper forward hand path trajectories **(B)**, as well as in the retraction **(E)**. **(C,F)** show that Lambda values in the forward reach and uninstructed retraction, respectively, are statistically similar for the comparison of illusory and proper hand path trajectories.

We found striking differences in movement trajectories across perceptual conditions according to the Wilk's lambda test, which we explain below. Mean trajectories are plotted in white with confidence intervals (colored tubes) for each point in the trajectory for veridical (green), illusory (blue), and proper (red) conditions for the intentional goal-directed, forward movement (Figure 6A) and the uninstructed, automatic retraction (Figure 6B). Note that each colored tube is representative of the collection of hand trajectories performed on each stimulus like the representative trajectory in Figure 5B. Since the unfolding of movement is critical in determining differences in approach, Wilk's lambda values are plotted based on the percentage of hand path trajectory completed (e.g., 25, 50, 75, and 100%) (Figure 7). Averaging these values across the entire path does not accurately represent the kinematics of the entire hand trajectory action loop, as shown by the curvature of each movement (Figure 6).

The analysis of the hand path trajectory using the Wilk's Lambda Test reveal a statistically significant difference between veridical and illusory conditions in the forward, goal-directed movement, as $\Lambda \leq [\Lambda {*}_{lower}:\Lambda {*}_{upper}]$ throughout the entire path's forward progression (Figure 7A). Recall that $\Lambda {*}_{lower}\;=\;\Lambda {*}_{\propto =0.05,p=3,vE=320,vH=2}=0.961$ and $\Lambda {*}_{upper}=\Lambda {*}_{\propto =0.05,p=3,vE=440,vH=2}=0.972$, as taken from Rencher (Rencher and Christensen, 2012). This behavior is also preserved in the non-instructed retraction (Figure 7D). As expected, the comparison between the veridical and proper conditions differs significantly in both the forward and retractory movements (Figures 7B,E). The illusory and proper conditions do not differ significantly in either movement class, as $\Lambda >[\Lambda {*}_{lower}:\Lambda {*}_{upper}]$ for all lambda values based on the percentage of path complete in both the forward and retraction cases (Figures 7C,F).

### Changes in hand orientation under different percepts

When examining the orientation of the hand as it approaches the target in each condition, hand-approach unit vectors in the veridical cases differ from those in the illusory and proper cases as shown by comparing the angles formed between each hand approach unit vector (Table 1), and the angles formed between each hand approach unit vector and the unit vector normal to the target's surface location (Table 2). Illusory and proper conditions produce similar hand postures when orienting toward the perceived target on the reverspective stimulus and the physical target for proper-perspective stimulus. The Kruskal-Wallis Test reveals a significant difference between angles formed by the illusory and proper hand approach unit vectors (∠ Illusory & Proper) vs. the angle formed between veridical and proper hand approach unit vectors (∠ Veridical & Proper) (Table 1). Note that illusory and veridical hand approaches are conducted on the same physical reverspective stimulus. No significant group differences were found for all other angle group comparisons (Table 1).

**Table 1:** **Kruskal-Wallis test for Angles between Unit Approach Vectors**.

**Angle group comparison**	*p*-value^*^
∠ Illusory & Proper^a^ vs. ∠ Veridical & Proper^b^	9.5608e-10 ± 6.8917e-14^*^
∠ Illusory & Veridical^c^ vs. ∠ Veridical & Proper	0.3288 ± 0.3607
∠ Illusory & Proper vs. ∠ Illusory & Veridical	0.0495 ± 0.1981

This table compares the angles formed (∠) by hand unit approach vectors in each condition using the Kruskal-Wallis Test. The Kruskal-Wallis Test is a non-parametric method (similar to an ANOVA) that uses ranks to determine whether or not samples come from the same distribution. When comparing ∠ Illusory & Proper vs. ∠ Veridical & Proper angle values, p = 9.5608e-10 ± 6.8917e-14. Values of p < 0.05 indicate significantly different groups. ∠ Illusory & Veridical vs. ∠ Veridical & Proper comparisons and ∠ Illusory & Proper vs. ∠ Illusory & Veridical comparisons do not reject the null hypothesis. This implicates that ∠ Illusory & Veridical vs. ∠ Veridical & Proper and ∠ Illusory & Proper vs. ∠ Illusory & Veridical are similar and belong to the same distribution.

a ∠ Illusory & Proper = $\left[{\hat{\text{I}}}_{1,\ldots 11}\right]\cdot \left[{\hat{\text{P}}}_{1,\ldots 11}\right]$, Illusory Unit Vectors · Proper Unit Vectors.

b ∠ Veridical & Proper = $\left[{\hat{\text{V}}}_{1,\ldots 11}\right]\cdot \left[{\hat{\text{P}}}_{1,\ldots 11}\right]$, Veridical Unit Vectors · Proper Unit Vectors.

c ∠ Illusory & Veridical = $\left[{\hat{\text{I}}}_{1,\ldots 11}\right]\cdot \left[{\hat{\text{V}}}_{1,\ldots 11}\right]$, Illusory Unit Vectors · Veridical Unit Vectors.

* Designates p < 0.05, indicating significant difference between groups.

**Table 2:** **Angle between Mean Hand Approach Unit Vectors and Target Surface Normals**.

**Target surface normal unit vector location**	**Mean hand approach unit vector**	**Mean Angle ± S.D. (degrees) between unit vectors**
Reverspective stimulus	Veridical	46.076 ± 16.101
	Illusory	84.008 ± 13.829
	Proper	86.314 ± 12.760
Proper-Perspective stimulus	Veridical	49.443 ± 17.016
	Illusory	18.377 ± 9.286
	Proper	17.772 ± 8.3623

The angles formed between the mean hand approach unit vector and the target surface normals on each stimulus are given in this table. The angle between the mean illusory hand approach unit vector and the actual unit vector normal to the target's location is nearly 90° (84.008° ± 13.829), whereas the mean veridical hand approach unit vector and the target surface normal produce an angle close to 45° (46.076° ± 16.101). Recall that the reverspective stimulus generates nearly 90° maximal differences between illusory and veridical perceptual states. When comparing the illusory hand approach unit vector to the proper-perspective target's surface normal, (which is representative of the illusory target surface normal if subjects act on the illusory geometry of reverspective stimulus), the mean angle is calculated as 18.377° ± 9.286. This may indicate that the hand orients toward the illusory target on the reverspective stimulus as it would do so on the proper-perspective stimulus, signifying a strong influence of the illusory percept on the motor action.

Next, we evaluated the angle formed between the mean unit approach vector and the unit vector normal to the target surface location in each condition (as illustrated in Figures 4A–C). Recall that the reverspective stimulus generates nearly 90° maximal differences between illusory and veridical perceptual states. The mean illusory hand approach unit vector and the actual unit vector normal to the target's location produce an angle close to 90° (84.008° ± 13.829), whereas the mean veridical hand approach unit vector and the target surface normal produce an angle close to 45° (46.076° ± 16.101) (Table 2). Although one would assume that the angle between the veridical hand approach unit vector and the target surface normal would be close to zero, the physical geometry of the reverspective stimulus induces obstacle-avoidance behavior, hindering the orientation of the right hand to act on the target normal to its location due to constraints on the arm's degrees of freedom. Proper hand approach unit vectors in relation to the proper-perspective target's surface normal produce an angle of 17.772° ± 8.362. When the illusory hand approach unit vector is compared to the proper-perspective target's surface normal, (which is representative of the illusory target surface normal if subjects act on the illusory geometry of reverspective stimulus), the mean angle is calculated as 18.377° ± 9.286. This result suggests that hand orientation toward the illusory target on the reverspective stimulus mimics hand orientations performed on the proper-perspective stimulus, indicating a strong influence of the Illusory percept on the motor trajectory.

### Normalized peak angular velocity distributions

**Figure 8:**
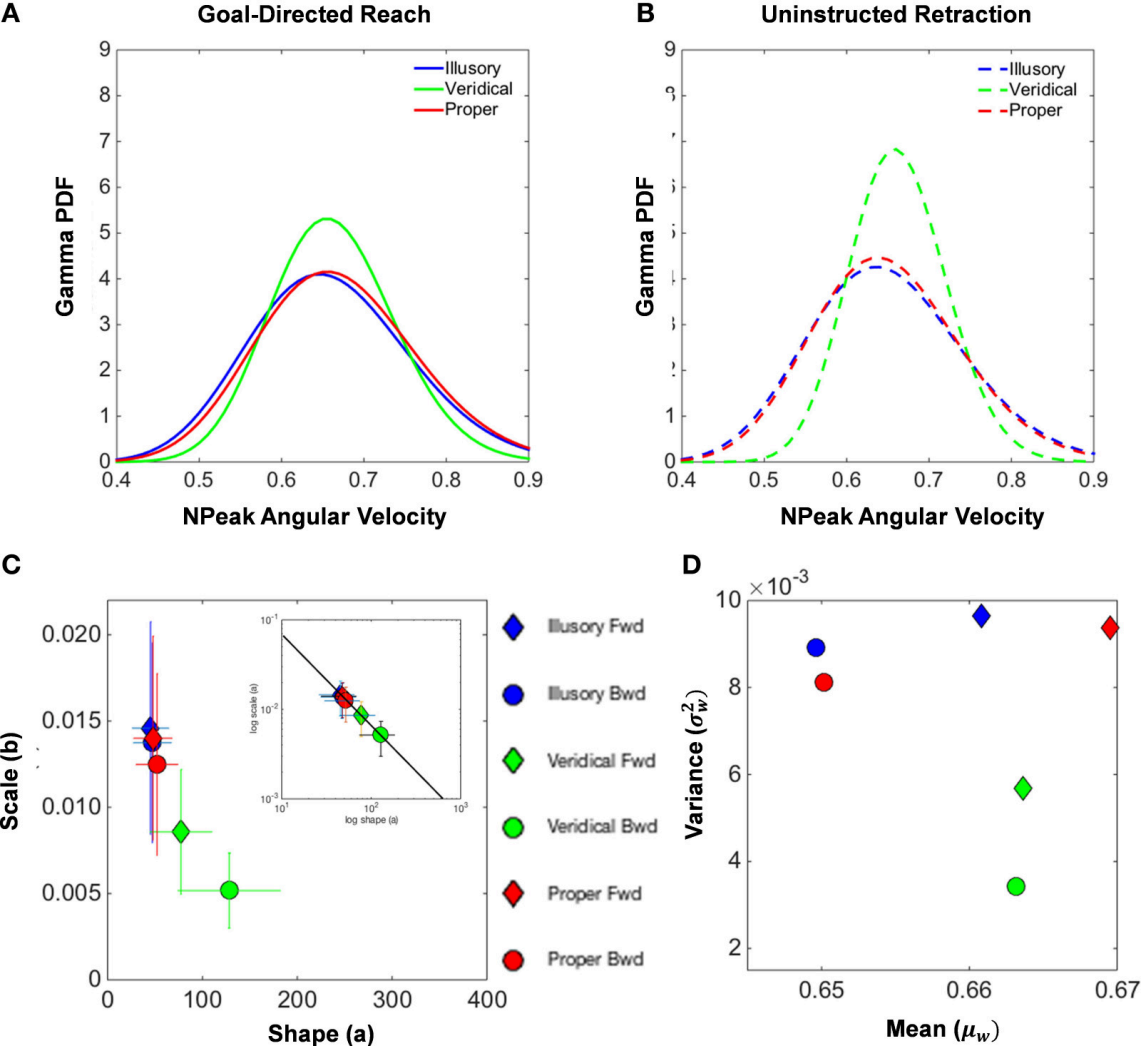
**Normalized peak angular velocity distributional analysis**. **(A,B)** illustrate the Gamma probability density function that is fitted to the underlying probability distribution of normalized peak angular velocities of the hand during the goal-directed reach **(A)** and the uninstructed retraction of the hand back to rest **(B)**. The illusory condition is shown in blue, the veridical in green, and the proper in red. The Gamma parameter place spanned by the shape (*a*) and scale (*b*) parameters for each empirically estimated Gamma function is shown in **(C)**. Filled diamonds designate forward goal-directed shape and scale parameters, and filled circles illustrate the supplemental retractions. Ninety-five percent confidence intervals from the MLE procedure are depicted by the crosshairs. The log-log plot of the Gamma plane is also shown to characterize the Gamma parameters by the exponential fit: *f*(*x*) = 0.638^*^x^−.992^, with coefficients given with 95% confidence intervals (see text for details on goodness of fit). The Gamma statistics, the mean (**μ**_**W**_) and the variance $\left(\sigma_{\text{W}}^{2}\right)$ parameters were estimated from the shape and scale parameters and plotted in the Gamma mean-variance parameter plane **(D)**.

Inspection of the underlying probability distribution of the normalized peak angular velocities for both the forward reach (Figure 8A) and uninstructed retraction (Figure 8B) reveals similarities between illusory and proper conditions. This figure summarizes the population data by pooling all individual results on the Gamma parameter plane (with confidence intervals for each point). The shape and scale parameters of the Gamma probability distribution exposes differences in the patterns of motor fluctuations in performance, illustrating a close clustering of the proper and illusory normalized peak angular velocity distributions in the upper left-hand corner, as opposed to the shape and scale parameters of the veridical distribution (Figures 8C,D).

We also uncovered a power law that governs the Gamma parameters of our model of motor-sensing behavior under perceptual state changes (inset in Figure 8). The empirically derived power law is given by *f(x)* = α^*^x^**β**^, with coefficients given with 95% confidence intervals: **α** **= 0.6387 [0.4475, 0.83]** for the intercept and **β = − 0.9923** [−**1.068**, −**0.9164**] for the slope. The goodness of fit for our model results in an R^2^ value of 0.9983, with the Sum of Squares due to Error (SSE) at a value of 1.205 ^*^ 10^−0^, and the Root Mean Square Error (RMSE) at 0.0001736.

As seen in the inset of Figure 8C, the log-log of the Gamma parameters segregates the veridical movement classes in contrast to the proper and illusory conditions. This governing power law shows how perceptual processes influence the speed-dependent statistics of motor trajectories. Specifically the shifts in the shape and dispersion of the estimated PDFs provide an index of how motor behavior is modulated by changes in internal mental states as evoked by illusory percepts. As the physical stimulus is veridical, the shape value of the PDF estimated from the moment by moment fluctuations in speed performance grows toward the Gaussian case and the noise-to-signal ratio drops. This results in an overall increase in the certainty of the motor program selection in response to a randomly presented percept. In contrast, the more ambiguous illusory stimuli result in lower certainty as captured by their systematically consistent increase in the noise-to-signal ratio and in the shape value decrement toward the more random Exponential case to the left of the shape axis.

In addition, Figure 8D reports the estimated moments mean (**μ**_***W***_) and variance $(\sigma_{\text{W}}^{2}$) for each condition and movement class. Note that the plots of the means and variances for the veridical conditions separate from the values found in the other conditions. This separation is most evident in the uninstructed automatic retractions, those that participants reportedly were unaware of. These findings also suggest that, under the veridical percept of the reverspective stimulus, tighter control of the angular rotations of the hand during the unfolding of movement is exhibited, whereas proper and illusory parameters tell a different story of higher uncertainty.

Based on the mean and variance estimated from the shape and scale parameters of the Gamma fitted probability density function, Table 3 calculates the index of dispersion via the Fano Factor, $\text{F}=\frac{\sigma_{\text{W}}^{2}}{\mu_{\text{W}}}$. Note that the Fano Factors (the noise-to-signal ratio) for normalized peak angular velocities are at their lowest in the veridical condition (0.0086 in the forward reach and 0.0052 in the retraction), whereas the proper and illusory Fano Factors are comparable and almost double these values in the forward case (0.0140 and 0.0146, respectively), and at their highest in the retraction (0.0125 and 0.0137, respectively). The veridical condition gives rise to more consistent movements with a trial-by-trial lower noise-to-signal ratio. Despite the random presentations of stimuli, when gathering the trials corresponding to each percept in the order in which they were presented, from trial to trial these percept-driven motions are also more predictable and reliable as they fall to the far right of the Gamma parameter plane and to the lowest scale value of all conditions. Recall that the scale value reflects the noise-to-signal ratio since the Fano Factor is the **variance/mean** = ***b***. These retracting veridical motions are under tight motor control despite being uninstructed, not pursuing an explicit goal, and the subject's reportedly not even being aware of the motion.

**Table 3:** **Fano factor calculations for the distribution of normalized peak angular velocities**.

**Condition**	**Goal-Directed reach**	**Uninstructed retraction**
Illusory	0.0096/0.6608 = 0.0146	0.0089/0.6496 = 0.0137
Veridical	0.0057/0.6636 = 0.0086	0.0034/0.6632 = 0.0052
Proper	0.0094/0.6695 = 0.0140	0.0081/0.6502 = 0.0125

The Fano Factor $\text{F}=\frac{\sigma_{\text{W}}^{\text{2}}}{\mu_{\text{W}}}$ measures the level of dispersion of each estimated Gamma probability distribution of the normalized peak angular velocities in each condition. The mean is designated as **μ**_W_, and the variance as $\sigma_{\text{W}}^{\text{2}}$ for time window w. Note how low variance values in the veridical cases (0.0057 in the forward reach and 0.0034 in the uninstructed retraction) are responsible for smaller Fano Factors (0.0086 and 0.0052, respectively), implicating that the joint rotations underlying the linear displacements of the hand in the veridical condition are under tighter control than that found in the proper and illusory conditions. Fano Factor values for proper and illusory conditions are comparable (0.0140 and 0.0146, respectively), and almost double the Fano Factors calculated for the veridical normalized peak angular velocity distributions. These findings suggest that the veridical condition gives rise to more consistent movements as the condition produces a trial-by-trial lower noise-to-signal ratio.

## Discussion

This work offers a new experimental paradigm and statistical platform to study perception and action loops in the psychological and neural sciences under a new personalized lens. Using this novel framework, we are able to characterize interactions with the illusory percept using the motor output fluctuations in performance that would be otherwise averaged and smoothed out as noise under the current “one-size-fits-all” approach to the kinematic variability analyses.

Through the personalized statistical approach we are able to unambiguously distinguish the illusory percept from its veridical counterpart, demonstrating the use of various movement biometrics to show how centrally driven top-down ventral stream processes impact sensory-motor performance of the peripheral nervous system. These changes can be immediately read out from stochastic movement signatures as the end-effector's action unfolds moment by moment.

In the spirit of psychophysical power laws relating sensation and perception (Stevens, 1957; Wolfe, 2009), here too we find a power law relation between the shape and the dispersion of the estimated distributions. As the value of the shape exponentially grows, the noise exponentially decays, thus sharpening the signal and revealing a tangible index of certainty needed to evaluate such tasks in laboratory settings. More specifically, the statistical characterization of the fluctuations in performance particular to each condition provides information on the level of certainty in the systems' decisions to select the appropriate motor program for the end effector among infinite possibilities that the arm affords. These fluctuations as the system continuously experienced the percepts are a form of kinesthetic sensory input that is not just passively sensed. Instead, the data clearly shows that the sensory motor systems selectively drive the use of the most adequate reach-to-grasp pattern for the illusory or for the physical geometry. In this sense this online, moment-by-moment information from the peripheral limbs integrated with the top-down visual percept helps anticipate the sensory consequences of impending actions and connects centrally driven mental awareness to peripheral bodily sensations. In the spirit of the active sensing paradigm proposed long ago (Von Holst and Mittelstaedt, 1950; Von Holst, 1954) here we have shown how to measure in an individualized way the statistical degree of how top-down visual information penetrates automatic motor performance, even when the performer is not fully unaware of the on-going motor processes.

In the past, psychophysical laws pertaining to the perception of external sensory stimuli have been subjectively estimated, relying on verbal reports made by subjects of the conscious recollection of perceptual experiences (Gazzaniga, 2012). Here, subjects internally generated movements when interacting with the illusory or veridical reverspective stimulus. Subjects, at times, consciously experienced this continuous stream of kinesthetic sensory input during the execution of deliberate motions toward the target. However, in the uninstructed retractions supplementing the goal-directed component of the action, subjects were reportedly largely unaware of the motor output fluctuations defining the kinesthetic sensory input. Surprisingly, these supplementary segments were by far the most informative in the veridical state when they experienced the actual physical percept. Their retraction movements had the lowest noise to signal ratio (lowest Fano Factor). As such, the noise-to-signal ratios of these transitional movements could tell us with high certainty, given the signatures of variability of the internal joint rotations of the moving hands, which trajectories most likely corresponded to the veridical percept and which were most likely from the illusory condition. The selective feature of these transitional motions and their statistical specificity may come as a surprise to some researchers, given that they are usually discarded as noise. However, they have been identified previously as providers of rich information in the sensory-motor domain (Torres, 2011, 2012, 2013a,b; Torres et al., 2014).

### Limitations of current study

A limitation of this study is that in each trial upon opening their eyes after the stimulus was placed in the subject's view, they were asked to report on the viewing of the middle building of each stimulus as either popping out or caving in. This is in contrast to allowing them to spontaneously report their percept. This verbal response may have influenced the motor performance. In future versions of this study we intend to refrain from asking participants to view the middle building of each stimulus as either popping out or caving in, allowing for spontaneous reporting of their percept. The rationale for not employing this strategy in the current version of the paradigm was that, since subjects were able to switch back and forth between each percept with minimal effort, we could then control for the number of trials per each condition. However, we note that even if they consciously experienced a different percept than they reported, the spontaneous retraction motions reflected the choice with high fidelity as well. Thus they will be the focus of future studies. Future work exploiting the spontaneous self-reporting of the experienced percept will be combined with the supplementary retracting motions. An investigation of whether or not a certain percept was favored over the other without the current constraints is also needed (see below).

### Future directions

By investigating natural behaviors using physically based (objective), automatic measures of motor performance, we provide new analytical tools that improve on existing methods by expanding our understanding of sensory-motor control mechanisms during perceptual tasks. Current work in our lab uses these metrics to integrate the time series of the signals from the body joints with those from electroencephalography in typical and atypical populations. Under the same personalized platform we may provide new insights into the pathophysiology of various disorders, shedding light on the connection between perceptual abnormalities tied to external stimuli and internal kinesthetic sensing. This may pave the way for a personalized comprehensive assessment of the systems' plasticity across central and peripheral nodes of an integrated network.

## Author contributions

All authors contributed to the study design. JN performed subject recruitment, testing, and data collection with the help of UM, JR. JR, UM designed the MATLAB program to run the experiment under the supervision of JN and TP. ET designed statistical analyses and provided the original MATLAB code to be adapted for the study. JN performed data analysis and interpretation under the close supervision of ET and TP. JN drafted the manuscript, and ET, PT provided critical revisions. All authors approved the final version of the manuscript for submission.

## Funding

The Nancy Lurie Marks Family Foundation Career Development Award to ET. This work was also supported by the NSF CDI Type I (Idea) Grant #094158 to EBT, the NSF GRFP Award #DGE-0937373 to JN, and the NIH Ruth L. Kirschstein NRS Award #T32-GM8339 from the NIGMS to JN.

### Conflict of interest statement

The authors declare that the research was conducted in the absence of any commercial or financial relationships that could be construed as a potential conflict of interest.
